# At‐Home Self‐Collection of Pharmacokinetic Data: Design and Results From a Phase 1 Open‐Label Feasibility Trial

**DOI:** 10.1002/cpdd.1495

**Published:** 2024-12-04

**Authors:** Arash Raoufinia, Susan E. Shoaf, Brian Rothman, Chelsea Ye, Chris Chung

**Affiliations:** ^1^ Otsuka Pharmaceutical Development & Commercialization Inc. Princeton NJ USA; ^2^ Verily South San Francisco CA USA

**Keywords:** attention‐deficit/hyperactivity disorder, clinical pharmacology, electrocardiograph, evidence‐based medicine, vital signs

## Abstract

Pharmacokinetic (PK) studies pose unique technical challenges. We present the design of a Phase 1, open‐label, fixed‐sequence, PK trial that aimed to compare the timing accuracy of participant‐ versus staff‐collected data, and we provide safety and tolerability outcomes for centanafadine treatment. Healthy adults aged 18‐55 years received a single 100‐mg centanafadine sustained‐release tablet at Visits 1, 2, and 4. PK samples (venous sampling and blood microsampling) and safety assessments (12‐lead electrocardiograms [ECGs] and vital signs) were collected by clinical site staff only at Visit 1. At Visit 2, site staff collected venous blood, and participants obtained blood microsamples, a 6‐lead ECG, and vital signs under staff supervision. At Visit 4, participants obtained blood microsamples, a 6‐lead ECG, and vital signs remotely. The absolute differences between actual and scheduled collection times for PK samples, ECGs, and vital signs are reported descriptively. Of the 20 participants, at least 75% obtained blood microsamples within 10 minutes of the planned nominal time. Absolute differences between actual and scheduled collection times of ECGs and vital signs were small. No adverse events were related to treatment. Overall, results support the feasibility of at‐home collection of PK samples, ECGs, and vital signs.

Traditional clinical trials are the gold standard for assessing the efficacy and safety of medical interventions. However, conventional clinical trials, conducted at centralized sites, may prove difficult for potential trial participants to reach and may contribute to recruitment difficulties resulting from requirements for on‐site participation.^1^ Recent advances in technology, including devices for at‐home collection of samples, digital devices for measuring physiological data, and digital platforms for engaging with patients, may enable researchers to conduct aspects of clinical trials remotely and with less involvement of clinical trial sites and their staff.^2^

However, pharmacokinetic (PK) studies pose unique technical challenges for remote data collection. PK samples are traditionally collected at specific time points by trained site staff. For a remote PK trial to be successful, participants need to be properly trained to collect samples within specific time windows and must learn how to store and ship samples according to procedures that ensure sample stability to avoid issues that can affect sample quality, such as extremes of temperature or humidity.^2^ At‐home PK studies could also add to patient burden if assessments are too frequent or too difficult to conduct.

We designed a trial to assess the feasibility of conducting clinical trials using at‐home self‐collection of PK samples, electrocardiogram (ECG) recordings, and vital signs in healthy adults, using centanafadine administration as an exemplar. Centanafadine is a first‐in‐class norepinephrine‐dopamine‐serotonin reuptake inhibitor^3^, ^4^ currently in development for the treatment of attention‐deficit/hyperactivity disorder in adults, children, and adolescents.^5^, ^6^ Various doses of centanafadine, ranging from 100 to 800 mg, have been studied, showing dose‐dependent adverse effects, with nausea, dizziness, headache, and decreased appetite being the most common (occurring in ≥10%) at the highest dose.^4^, ^7^

Here, we present the design and results of a Phase 1, open‐label, fixed‐sequence trial detailing the timing accuracy of at‐home sample collection by participants after administration of centanafadine compared with that of sample collection performed by trial site staff.

## Methods

### Trial Design

This was a Phase 1, open‐label, fixed‐sequence trial conducted at a single site in the United States (Diablo Clinical Research, Walnut Creek, CA) between April 23, 2021, and July 9, 2021. The trial consisted of a screening period, 2 clinic visits (Visits 1 and 2), 2 at‐home visits (Visits 3 and 4), and a follow‐up clinic visit (Visit 5) (Figure S1).

A 100‐mg dose of centanafadine was selected, as the safety and tolerability in healthy adults for total daily doses up to 800 mg had been established.^4^, ^7^ Initial absorption is rapid; peak concentrations are reached at about 2 hours; and elimination half‐life is short, about 4 hours. A single 100‐mg tablet was expected to be easy to administer, produce concentrations well above the lower limit of quantitation (5.00 ng/mL), and have a safe profile. Additionally, concordance between centanafadine concentrations in blood collected using a fully validated volumetric absorptive microsampling (VAMS) technique and venous plasma concentrations had been established.

The trial was conducted in accordance with the International Council for Harmonization Good Clinical Practice guidelines, the Declaration of Helsinki, the Council for International Organizations of Medical Science recommendations, and US Food and Drug Administration regulations. The informed consent form, protocol, and amendments for the trial and participant compensation were approved by the central institutional review board (WCG IRB). Prior to the start of trial procedures, participants used an electronic member portal to review the trial consent video and informed consent form. After discussing their understanding of the information with site staff, participants provided informed consent in the portal, using their full legal name and an electronic representation of their signature.

### Participants and Treatment

Participants were generally healthy adults aged 18‐55 years, with a body mass index between 19.0 and 32.0 kg/m^2^. Full inclusion and exclusion criteria for the study are noted in Table S1. General health status was determined by medical history, physical examination, a 12‐lead ECG, and laboratory assessments (serum/urine chemistry, hematology, and serology). Participants received a single 100‐mg centanafadine sustained‐release (SR) tablet at Visits 1, 2, and 4, administered with approximately 240 mL of water after a fast of at least 8 hours. Participants were to abstain from drinking water for 1 hour before dosing and abstain from food and fluids for 2 hours after dosing to ensure consistent dosing in the fasted state.

### Trial Schedule and Procedures

#### Schedule

At the screening visit, which occurred from Day −21 to Day −1 (Figure S1), candidates registered online using a web‐based clinical trial management platform (Clinical Research Suite, Verily), and informed consent was obtained electronically. Site staff confirmed the inclusion and exclusion criteria with trial participants and assessed and recorded baseline data.

Prior to any protocol‐related procedures at Visit 1, each participant received a set of mobile medical devices, including a Model IR20b ear thermometer (Foracare, Inc.) and a Model UA‐651 upper‐arm blood pressure monitor (A&D Medical) that enabled the collection and transmission of data to the trial site wirelessly. Participants also received a KardiaMobile 6‐lead ECG device (AliveCor) and blood microsample kits (Neoteryx) containing a Mitra microsampling device that used VAMS technology, a lancet, cotton gauze, a bandage, and a sample label. Site staff loaded the clinical trial app and the ECG device app (along with the associated ECG data‐analyzing portal registration) on each participant's mobile phone. Test video visits were conducted by site staff to train participants on the remote trial procedures and to supervise participants while they obtained their own ECG recordings; these visits lasted as long as necessary to complete all the checks and procedures. The clinical trial platform provided in‐app notifications about upcoming procedures to be performed and allowed the participant to confirm completed procedures.

Visit 1 was similar to the experience of traditional Phase 1 single‐dose PK trials, including administration of the first treatment dose of centanafadine and collection of traditional venous blood and microsamples by the staff (before dosing and then at 2, 4, and 6 hours after dosing), ECG recordings (12‐lead ECG obtained by staff, 6‐lead ECG obtained by participants), and vital signs (obtained by staff). Venous blood was to be collected before the blood microsamples, per the protocol. Additionally, the Columbia Suicide Severity Rating Scale (C‐SSRS) was completed, as centanafadine is being developed for a psychiatric indication.

Participants reported for Visit 2 at least 48 hours but no more than 14 days after Visit 1. At this visit, participants self‐administered centanafadine and obtained their own vital signs, 6‐lead ECG, and PK microsamples under staff supervision; the C‐SSRS was also obtained. During this visit, staff provided verbal instructions or physical assistance to enable participants to complete the planned assessments. Retraining (up to 3 times per assessment) could be provided, and the need for intervention and retraining was formally documented. Site staff also recorded whether participants were able to complete each trial procedure, and any participant who failed to successfully self‐collect samples and measurements following 3 retraining sessions was to be discontinued from the trial.

Visit 3, which occurred any time after Visit 2 and within 72 hours prior to Visit 4, was the first at‐home visit in the trial completed via the trial app. During the video visit, the site staff confirmed the participant's possession of centanafadine SR, confirmed setup of remote monitoring devices, and collected information on adverse events (AEs) and concomitant drugs. No venous blood, microsampling, vital sign, or ECG collections occurred at Visit 3. Visit 4, the second at‐home visit in the trial, occurred at least 48 hours but no more than 14 days after Visit 2. Participants self‐administered centanafadine at home during a video chat with site staff via the trial app and obtained their own PK microsamples, 6‐lead ECG, and vital signs; the C‐SSRS assessment was obtained for a final time. Participants could also contact site staff via the video link or by telephone if they encountered difficulties with self‐collection procedures. The site staff contacted the participants after dosing at Visit 4 to record any AEs. Participants returned on‐site for Visit 5, the final follow‐up visit, within 7 (+2) days after Visit 4. End‐of‐trial assessments conducted by site staff included physical examination, vital sign measurement, a 12‐lead ECG recording, and laboratory studies.

#### Microsampling Procedure

VAMS methodology has been increasingly used in recent years to conduct PK studies with fixed blood volumes; this method provides an alternative to the dried blood spot technique, as it overcomes the limitations of sample nonhomogeneity, the effects of hemocrit levels on sample size/spread, and environmental degradation.^8^ In studies reported to date, VAMS has shown good correlations with conventional venous sampling.^9^ VAMS has been validated for the measurement of multiple pharmacologic agents, and its use in an at‐home setting for routine clinical care has been shown to be feasible.^10^, ^11^, ^12^, ^13^, ^14^ For this trial, microsamples were obtained using a Mitra VAMS device manufactured by Neoteryx (now Trajan). Collection using the Mitra device was performed by touching the absorptive tip of the device to a drop of blood created via lancet puncture of the finger. After collection, the tip was dried under desiccant and shipped to a bioanalytical laboratory for analysis. Further details of the sampling procedures and PK analyses can be found in Supplemental Information Text S1.

#### Trial Outcomes

Outcomes of interest for this analysis were comparisons of actual versus scheduled times for collection of venous PK samples and microsamples, ECG recordings, and vital signs. Safety and tolerability were assessed by treatment‐emergent adverse events (TEAEs), clinical laboratory tests, ECGs, vital signs, physical examinations, and suicidality (via the C‐SSRS).

### Statistical Analysis

Descriptive statistics (mean, standard deviation, median, minimum, maximum, 25th percentile, and 75th percentile for the absolute difference) were provided by visit for deviations of actual collection times of PK samples; ECG recordings; and vital signs from the scheduled times at Visits 1, 2, and 4. Statistical calculations were conducted using SAS version 9.4 (SAS Institute).

## Results

### Participant Demographics and Baseline Characteristics

In total, 23 individuals were screened, and 20 were enrolled. Participants had a mean (standard deviation) age of 35.9 (11.1) years, and 18 (90%) were female. Additional details are reported in Table S2. All enrolled participants completed the trial.

### Pharmacokinetic Sample Collection

The absolute differences (third quartile) between the actual and scheduled collection times for venous PK samples were 1.5‐3 minutes (Visit 1) and 1‐1.5 minutes (Visit 2). The absolute median differences between the actual and scheduled collection times of PK microsamples were 5‐7 minutes (Visit 1), 4 minutes (Visit 2), and 2‐3 minutes (Visit 4). The third quartile absolute differences for blood microsamples were in the range of 5‐8 minutes at Visit 4, similar to the ranges of 8.5‐10 and 6‐7.5 minutes at Visits 1 and 2, respectively (Table 1). Thus, at least 75% of participants had obtained blood microsamples within 10 minutes of the planned nominal time. Individual differences between the actual and scheduled collection times of PK samples ranged from 0 to 44 minutes for venous samples and from 0 to 100 minutes for microsamples.

**Table 1:** Absolute Differences Between Actual and Scheduled Times for Pharmacokinetic Sample Collection (in Minutes)

	Visit 1^a^			Visit 2^b^			Visit 4^c^		
Sample type	2 hours (n = 20)^d^	4 hours (n = 20)	6 hours (n = 20)	2 hours (n = 20)	4 hours (n = 20)	6 hours (n = 20)	2 hours	4 hours	6 hours
Plasma venous samples									
Median	0	0	0	0	0	0	–	–	–
IQR	0‐3	0‐1.5	0‐3	0‐1	0‐1.5	0‐1	–	–	–
Blood microsamples									
Median	7	5.5	5	4	4	4	2	3	3
IQR	4.5‐9	3.5‐10	3.5‐8.5	2.5‐6.5	2.5‐6	2.5‐7.5	1‐5	1‐8	1‐5.5

IQR, interquartile range (ie, quartiles 1‐3).

a Plasma venous samples and blood microsamples were collected by staff at Visit 1.

b Plasma venous samples were collected by staff and blood microsamples by participants under staff supervision at Visit 2.

c Blood microsamples were collected by participants at home at Visit 4.

d Plasma venous samples (n = 19) collected at Visit 1, 2 hours after dosing. The collection date and time for 1 participant were recorded before the dosing date and time; therefore, it is shown as collected at screening and not at Visit 1, Hour 2.

### Electrocardiogram Readings

The mean absolute differences between the actual and scheduled times of ECG recordings were small, indicating that ECGs were obtained close to the nominal time point. Mean differences were between 1.4 and 8 minutes (Table 2); individual differences ranged from 0 to 63 minutes.

**Table 2:** Absolute Differences Between Actual and Scheduled ECG Recording Times (in Minutes)

	1 hour after dosing		3 hours after dosing		5 hours after dosing	
Parameter	n^a^	Mean (SD)	n^a^	Mean (SD)	n^a^	Mean (SD)
**12‐lead standard**						
Visit 1						
ECG heart rate, mean	19	6.8 (3.6)	19	5.3 (2.6)	20	6.3 (2.9)
PR interval, aggregate	19	–	19	–	20	–
QRS duration, aggregate	19	–	19	–	20	–
QT interval, aggregate	19	–	19	–	20	–
QTcB interval, aggregate	19	–	19	–	20	–
QTcF interval, aggregate	19	–	19	–	20	–
RR interval aggregate	19	–	19	–	20	–
**6‐Lead standard**						
Visit 1						
ECG heart rate, mean	20	2.2 (2.5)	20	5.7 (13.4)	19	2.3 (2.7)
PR interval, aggregate	20	–	20	–	19	–
QRS duration, aggregate	20	–	20	–	19	–
QT interval, aggregate	20	–	19	–	17	–
QTcB interval, aggregate	20	–	19	–	17	–
QTcF interval, aggregate	20	–	19	–	17	–
RR interval, aggregate	20	–	20	–	19	–
Visit 2						
ECG heart rate, mean	20	3.7 (5.2)	20	1.5 (1.6)	20	1.4 (2.8)
PR interval, aggregate	19	–	20	–	20	–
QRS duration, aggregate	20	–	20	–	20	–
QT interval, aggregate	20	–	20	–	20	–
QTcB interval, aggregate	20	–	20	–	20	–
QTcF interval, aggregate	20	–	20	–	20	–
RR interval, aggregate	20	–	20	–	20	–
Visit 4						
ECG heart rate, mean	19	6.1 (7.0)	20	8.0 (14.0)	20	7.8 (8.9)
PR interval, aggregate	18	–	20	–	20	–
QRS duration, aggregate	18	–	20	–	20	–
QT interval, aggregate	18	–	18	–	19	–
QTcB interval, aggregate	18	–	18	–	19	–
QTcF interval, aggregate	18	–	18	–	19	–
RR interval, aggregate	19	–	20	–	20	–

ECG, electrocardiogram; QTcB, QT interval corrected for heart rate using the Bazett formula; QTcF, QT interval corrected for heart rate using the Fridericia formula; SD, standard deviation.

a Participants with valid assessment at time point.

### Vital Signs

The mean absolute differences between the actual and scheduled collection times of vital signs were small, indicating that vital signs were obtained close to the nominal time point. Mean differences were between 3.9 and 10.1 minutes (Table 3); individual differences ranged from 0 to 67 minutes.

**Table 3:** Absolute Differences Between Actual and Scheduled Vital Sign Collection Times (in inutes)

	1 hour after dosing		3 hours after dosing		5 hours after dosing	
Parameter	n^a^	Mean (SD)	n^a^	Mean (SD)	n^a^	Mean (SD)
Visit 1						
Blood pressure^b^, supine	20	8.6 (3.1)	20	7.1 (2.7)	19	7.5 (2.6)
Heart rate, supine	20	8.6 (3.1)	20	7.1 (2.7)	19	7.5 (2.6)
Temperature	20	10.1 (7.5)	20	5.6 (3)	18	6.7 (3.9)
Visit 2						
Blood pressure^b^, supine	20	7.4 (3.7)	20	5 (2.1)	20	5.2 (2.0)
Heart rate, supine	20	7.4 (3.7)	20	5 (2.1)	20	5.2 (2.0)
Temperature	20	6.6 (4.7)	20	3.9 (2.6)	20	4.3 (2.6)
Visit 4						
Blood pressure^b^, supine	20	7 (6.4)	20	9.5 (13.8)	19	6.3 (5.5)
Heart rate, supine	20	7 (6.4)	20	9.5 (13.8)	19	6.3 (5.5)
Temperature	20	7 (6.5)	20	10.1 (14.1)	18	5.9 (6.2)

SD, standard deviation.

a Participants with data available for the calculation of absolute difference.

b As systolic and diastolic measurements were taken at the same time, outcome results were identical for both.

### Safety and Tolerability

Only 2 TEAEs occurred; both were mild (injection site [venipuncture] extravasation and bone pain), and none were judged to be related to centanafadine. No TEAEs led to discontinuation, and no serious TEAEs or deaths occurred. No clinically relevant changes in laboratory values, ECGs, or vital signs were observed during the trial. C‐SSRS assessments indicated that no participants had suicidal ideation or behavior during the trial.

## Discussion

In this exploratory trial, we compared the timing accuracy of at‐home self‐collection of PK, ECG, and vital sign data for participating adults versus that of the same data collected by trial site clinical staff. The results of the current trial indicate that with proper training, participants were able to collect PK and safety data remotely. The absolute median time between the actual and scheduled collection times for PK microsamples at all 3 visits was higher (2‐7 minutes) than that for venous samples collected by clinic staff (1‐3 minutes), likely because blood microsamples were collected after venous samples during Visits 1 and 2 as per protocol, leading to some blood microsample collections falling out of the 5‐minute window. Despite the unique technical challenges to remote data collection, these results indicate that at‐home patient‐collected data can be obtained successfully.

As expected, the 100‐mg dose of centanafadine SR chosen for this trial was well tolerated, with no treatment‐related TEAEs being reported among the healthy adult participants. The trial results also show that participants are able to conduct certain safety assessments remotely. Most vital signs and 6‐lead ECG recordings were obtained within the 15‐minute collection window. Although certain limitations of the 6‐lead ECG (such as in diagnosing ischemia and measuring QT intervals) prevent it from fully replacing its 12‐lead counterpart,^15^ the ability of participants to successfully obtain 6‐lead ECG data at home may reduce the frequency of more expensive 12‐lead ECG monitoring via in‐clinic visits.

Altogether, the results of this trial support remote data collection as a supplement to traditional in‐clinic safety monitoring. Remote self‐collection of data can provide several advantages for researchers and clinical trial participants. These advantages may include the ability to collect data at additional time points, increased access to individuals who would otherwise be unable to participate in traditional site‐based clinical trials, and diversification of patient participation.^1^ However, it should also be noted that the current trial may also be subject to bias (through self‐selection), due to the types of individuals who choose to participate. Prior analyses have indicated that people who have time to attend clinic visits (eg, are unemployed, homemakers, or retired), those with low incomes (and in need of the financial incentives commonly offered), or those with a high level of curiosity and persistence may be more likely/willing to take part in early‐phase clinical trials.^16^, ^17^ With more diversified enrollment, particularly in terms of sex, our trial results may be more generalizable to real‐world treatment situations. Finally, at‐home data collection in clinical trials may reduce burdens associated with time and travel, given that trial location is one of the most important challenges to overcome for people to participate in clinical trials.

## Conclusion

In this Phase 1 PK trial, centanafadine SR 100 mg was safe, with no treatment‐related AEs. Trial participants demonstrated their ability to successfully obtain blood microsamples, ECGs, and vital sign data at home. Although traditional clinical trials are the gold standard for assessing the efficacy and safety of medical interventions, this trial shows that recent advances in technology can be leveraged to conduct aspects of clinical trials remotely with less involvement of clinical trial sites and their staff.

## Conflicts of Interest

Arash Raoufinia, Susan Shoaf, Brian Rothman, and Chris Chung are employees of Otsuka Pharmaceutical Development & Commercialization, Inc. Chelsea Ye is an employee of Verily, which received funding from Otsuka for the clinical trial management platform used in this trial.

## Funding

This study was funded by Otsuka Pharmaceutical Development & Commercialization, Inc.

## Supporting information



Supporting Information
