# Supplementary material for: At‐Home Self‐Collection of Pharmacokinetic Data: Design and Results From a Phase 1 Open‐Label Feasibility Trial
Source: Clin Pharmacol Drug Dev. 2024 Dec 4;14(1):11–7. doi: 10.1002/cpdd.1495 (PMC11701959; doi:10.1002/cpdd.1495)
Supplement: Supplementary file 1 — Supporting Information [file CPDD-14-11-s001.pdf]

SUPPLEMENTAL DIGITAL CONTENT

Text S1. Additional Pharmacokinetic (PK) Procedures and Outcomes

Samples were stored until shipment (plasma samples at -80°C [± 20°C] or on dry ice; microsamples at room temperature in a bag containing desiccant), and in-clinic samples were shipped in batches for analysis. The site was provided with pre-labeled bags for in-clinic sample sorting and shipment, and participants were provided with prepaid UPS packaging for at-home sample shipment from their residence. Alternatively, at-home samples could be deposited at the site at the next scheduled in-clinic visit and shipped from there. All microsamples, regardless of location, were handled identically, being placed into a bag containing desiccant at room temperature (15ºC–30ºC) until shipment.

A total of 160 plasma samples were received by the bioanalytical laboratory. Concentration results for centanafadine were generated for all 160 samples. A total of 240 dried whole blood samples were received, and concentration results for centanafadine were generated for 206 samples. The remaining 34 samples (27 postdose and 7 predose) were not analyzed due to undersampling. The high performance liquid chromatography-tandem mass spectrometry method used for plasma and blood analyses had adequate linearity, specificity, sensitivity, precision, and accuracy.

Although this trial was designed to assess the feasibility of at-home sample collection, rather than an analysis of centanafadine PK measurements, we conducted Deeming regressions of natural log-transformed microsampling versus venous plasma concentrations for centanafadine. At visit 1, the slope was 1.047, and at visit 2 it was 1.001. The 95% confidence intervals at both visits included 1; thus, linearity was concluded.

Table S1. Inclusion and Exclusion Criteria

**Inclusion criteria**	• Male or female aged 18–55 years<br>• Body mass index 19.0–32.0 kg/m^2<br>• In good health based on:<br>– Medical history<br>– Physical examination<br>– ECG<br>– Serum/urine chemistry, hematology, and serology tests<br>• Ability to provide written informed consent prior to initiation of any trial-related procedures, and the ability, in the opinion of the principal investigator, to comply with all requirements of the trial
**Exclusion criteria**	• Women who were breastfeeding and/or had a positive pregnancy test result prior to receiving trial drug<br>• Sexually active men or women of childbearing potential or their partners who did not agree to practice 2 different approved methods of birth control or remain fully abstinent during the trial and for 30 days after the last dose of trial drug (periodic abstinence [eg, calendar, ovulation, symptothermal, or postovulation methods] or withdrawal was not an acceptable method of contraception). If employing birth control, 2 of the following methods were required:<br>– Vasectomy, tubal ligation, intrauterine device, birth control pill, birth control implant, birth control depot injection, birth control patch, condom with spermicide, sponge with spermicide, or occlusive cap (vaginal diaphragm or cervical/vault cap) with spermicide<br>– Consensual sexual activity that could not biologically result in pregnancy may not have been subject to required birth control methods, following discussion with the medical monitor<br>• Male participants were also required to not donate sperm from trial screening through 30 days after the last dose of trial drug<br>• Clinically significant abnormality in past medical history or physical examination that, in the investigator's or sponsor's opinion, may have placed the individual at risk or interfere with outcome variables, including absorption, distribution, metabolism, and excretion of the trial drug. This included, but was not limited to, history of or concurrent cardiac, hepatic, renal, neurologic, endocrine, gastrointestinal, respiratory, hematologic, dermatologic, or immunologic disease

	• History of drug and/or alcohol use disorder within 2 years prior to screening, based on the investigator's judgment<br>• History of or current hepatitis, human immunodeficiency virus, or acquired immunodeficiency syndrome, or carriers of hepatitis B surface antigen and/or hepatitis C antibodies; exceptions were permitted for participants with a prior history of infection with hepatitis A who had fully recovered and were experiencing no liver sequelae<br>• History of any medically significant drug allergy or known or suspected hypersensitivity<br>• A positive urine or breath alcohol test and/or urine drug screen for substances of abuse at screening or at visit 1<br>• Participants who took an investigational drug within 30 days prior to screening<br>• Any history of significant bleeding or hemorrhagic tendencies<br>• A history of difficulty in donating blood<br>• Participants without a permanent physical residence<br>• Consumption of alcohol within 72 hours prior to the first dose of trial drug<br>• Use of prescription drugs, over-the-counter drugs, herbal remedies, or vitamin supplements within 14 days prior to the first dose of trial drug, and/or antibiotics within 30 days prior to the first dose of trial drug; the sponsor could allow exceptions only if the drug's administration was deemed unlikely to impact the PK results<br>• Exposure to any substances known to stimulate hepatic microsomal enzymes within 30 days prior to the first dose of trial drug (eg, occupational exposure to pesticides, or organic solvents)<br>• Use of tobacco products or daily exposure to second-hand smoke within 2 months prior to screening, or urine cotinine concentrations >200 ng/mL, or serum cotinine concentrations >20 ng/mL at screening or at visit 1<br>• Uncontrolled hypertension, defined as supine SBP ≥140 mmHg and/or supine DBP ≥90 mmHg at screening or at visit 1, or symptomatic hypotension or orthostatic hypotension, defined as a decrease of ≥20 mmHg in SBP and/or a decrease of ≥10 mmHg in DBP after ≥3 minutes of standing compared with the previous supine SBP at screening or at visit 1

	– The blood pressure measurement could be repeated after 5 minutes, up to a total of 3 times, and eligibility was determined based on the last measurement<br>• Participants who had a supine heart rate, after resting for ≥3 minutes, outside the range of 50–90 BPM; the sponsor could allow exceptions if the results were not considered to be clinically significant<br>• History of serious mental disorders that, in the opinion of the investigator, would have excluded the person from participating in this trial<br>• Participants who answered "Yes" on the C-SSRS Suicidal Ideation Item 4 (Active Suicidal Ideation with Some Intent to Act, Without Specific Plan) within the 12 months prior to screening or at visit 1, OR participants who answered "Yes" on the C-SSRS Suicidal Ideation Item 5 (Active Suicidal Ideation with Specific Plan and Intent) within the 12 months prior to screening or at visit 1, OR participants who answered "Yes" on any of the 5 C-SSRS Suicidal Behavior Items (actual attempt, interrupted attempt, aborted attempt, preparatory acts, or suicidal behavior) within the 24 months prior to screening or at visit 1, OR participants who, in the opinion of the investigator, presented a serious risk of suicide<br>• Participants with previous exposure to centanafadine<br>• Any participant who, in the opinion of the investigator, should not participate in the trial<br>• Participants who were unwilling to perform blood sampling using the microsampling device<br>• Participants who were unwilling to use any of the trial devices or technologies<br>• The donation of blood or plasma within 30 days prior to the first dose of trial drug<br>• Abnormal ECG findings at screening or visit 1, as follows:<br>– QTcF >450 msec for male participants or >470 msec for female participants<br>– QRS interval >120 msec<br>– PR interval >200 msec<br>• History of unexplained syncope

BP, blood pressure; BPM, beats per minute; C-SSRS, Columbia Suicide Severity Rating Scale; DBP, diastolic blood pressure; ECG, electrocardiogram; PK, pharmacokinetic; QTcF, QT interval corrected for heart rate using Fridericia formula; SBP, systolic blood pressure.

Table S2. Participant Demographics and Baseline Characteristics

Characteristic	Enrolled participants (n=20)
Age (years), mean (SD)	35.9 (11.1)
Female sex, n (%)	18 (90.0)
Body mass index (kg/m^2), mean (SD)	25.6 (2.9)
Race, n (%)	
White	12 (60.0)
Black	3 (15.0)
Asian	3 (15.0)
Other races[a]	2 (10.0)
Hispanic ethnicity, n (%)	7 (35.0)

SD, standard deviation.
[a]Includes American Indian or Alaska Native.

Figure S1. Schematic of the Trial Design

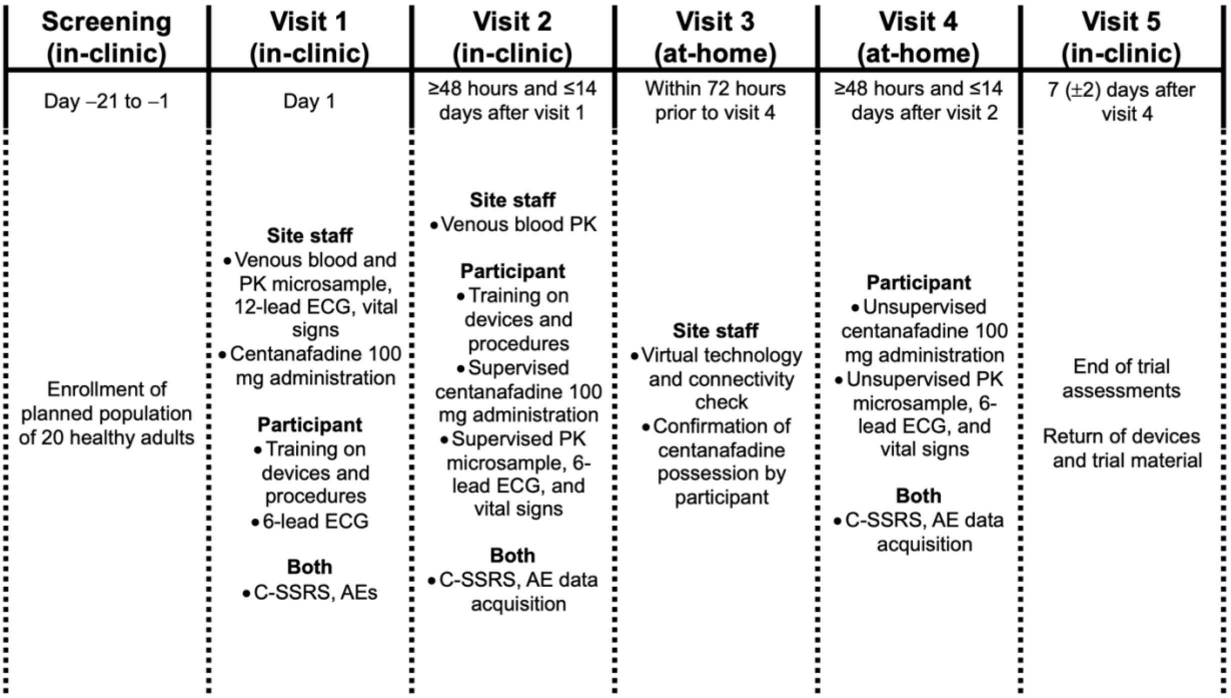

AE, adverse event; C-SSRS, Columbia Suicide Severity Rating Scale; ECG, electrocardiogram; PK, pharmacokinetics.
